# Income inequities in end-of-life health care spending in British Columbia, Canada: A cross-sectional analysis, 2004-2006

**DOI:** 10.1186/1475-9276-10-12

**Published:** 2011-03-16

**Authors:** Colleen M Cunningham, Gillian E Hanley, Steven G Morgan

**Affiliations:** 1 Centre for Health Services and Policy Research, University of British Columbia, 201 - 2206 East Mall, Vancouver B.C., V6T1Z3 Canada; 2 School of Population and Public Health, University of British Columbia, 201 - 2206 East Mall, Vancouver B.C., V6T1Z3 Canada

## Abstract

**Background:**

This study aimed to measure the income-related inequalities and inequities - the inequalities that remain after accounting for differences in health need - in expenditure on fully publicly covered (hospital and ambulatory) and partially publicly covered (prescription drugs) services for those in their last year of life in the province of British Columbia (B.C.), Canada. We focused on a decedent population for three reasons: to minimize unmeasured need differences among our cohort and therefore isolate income effects; to explore inequities for a high-spending window of health care use; and, because previous studies have found conflicting relationships between income and decedent health care spending, to further quantify this relationship.

**Methods:**

We used linked administrative databases to describe spending on health services by income for all 58,820 deaths of B.C. residents 65 and older from 2004 to 2006. Regression analyses examined the association between income and health care spending, adjusting for age, sex, health status, cause of death, and other relevant factors. We then used concentration indexes to measure both inequalities and inequities separately for three key types of services. Analyses were also run separately for men and women.

**Results:**

On average, per capita expenditure on acute health care in the last year of life was $20,705 (CDN2006). In need-adjusted regression analyses, we found decedents in the highest income quintile had 11% lower hospital expenditures, 15% higher specialist expenditures and 23% higher prescription drug expenditures than decedents in the lowest income quintile. Concentration index analysis suggested that spending for all types of care was concentrated among those with higher income before adjusting for need. Need-adjusted equity results mirrored regression findings and suggested patterns of inequities that were more pronounced among male decedents than females.

**Conclusions:**

Despite the universal health care system in B.C., we found patterns of inequity in spending by income in the last year of life, even for fully publicly covered services. These results, parallel to relationships between income and spending from previous studies of the B.C. population, suggest persistent income-related inequities in the health care Canadians receive throughout their lives.

## Background

Equity in access to health care, or equal access to care for those with equal need, has long been a concern among health services researchers and is a primary motivation for universal health insurance systems. Because use of and spending on health care are largely influenced by need for care, and health status varies by income [1,2], differences by income in expenditure on health care, or inequalities in spending, are not necessarily problematic. However, inequalities that remain after accounting for differences in need, or inequities in use of health care, signal potential inconsistencies between actual health care use or access to care and policy ideals.

Previous research has found that income-related differences in the use of health services, both inequalities and inequities, appear to exist in countries with (e.g. Canada and most countries in Europe) and without (e.g. the U.S.) universal health coverage [3]. Recent studies from the U.S., Europe, and Canada that have controlled for differences in need using self-reported health status (usually measured on a five-point scale from excellent to poor) along with either self-assessed disease burden or activity limitations have found evidence of significant inequities across many health care services [4-6]. For example, a study based on 1998-2001 non-elderly respondents to the U.S. National Health Interview Survey found use of ambulatory care to be inequitable, with those of higher income using more services than need would predict [4]. In comparing use of physicians across 12 European countries using pan-European survey data, van Doorslaer et al. (2004) found pro-poor inequities in visits to GPs and pro-rich inequities in visits to specialists in virtually all countries [5]. For Canada, using data from the 2003 wave of the Canadian Community Health Survey (CCHS), Allin (2008) found pro-rich inequities in specialist visits, and for probability of a visit to a GP, and pro-poor inequities in inpatient care for Canada, but no evidence of income inequities in total visits to GPs or for inpatient care separately for the province of B.C. [7]. A recent study using administrative claims databases for the full population of B.C., which used diagnosis code based case-mix tools to adjust for health status, corroborated the CCHS-based findings of pro-rich inequities in specialist care, but found pro-poor inequities in GP visits and inpatient care [8]. While the relationship between income and prescription drug use has been studied extensively, little work has been done to directly measure inequities. In sum, while there appears to be some consensus regarding pro-rich inequities for specialist visits, the trends in inequity findings for other acute care services, even those specific to B.C., are less clear.

Reviewers and authors of past studies of equity in health care argue that a key methodological limitation in constructing measures of inequity lies in the difficulty in adequately controlling for variations in need, both in survey and administrative data based studies [4,8,9]. The inability to properly measure and control for differences in need for health care could imply bias in previous measures of inequity.

We aimed to measure and compare income-related inequities in the use of medical services, hospital care, and prescription drugs in B.C. in the last year of life. We focused on end-of-life care for a cohort of seniors (age 65 and older) as this allowed for much greater need standardization than the research that has examined the entire population. End-of-life cohorts have been used by Wennberg, Fisher and colleagues to help reduce bias in the measurement of regional variations in health services use and cost [10-12]. As such, they also appear to be good candidates for investigating socioeconomic disparities. As well, this is an important window of health care use, and of increasing policy importance as baby-boomer populations in Canada and other developed countries approach old age and policies surrounding end-of-life care gain prominence [13].

Previous studies of income-related differences in use of health care at end-of-life have found mixed results. Studies of U.S. Medicare populations have found weak negative relationships between area-level income and health care spending [14-16], while studies of Swiss and Swedish residents found spending on health care at end of life to increase with household-level income [17,18]. Thus, we also aimed to further explore the end-of-life spending and household income relationship by examining all forms of acute health care spending together and separately by type, among decedents 65 and older.

As supplementary analyses, we performed sex-disaggregated regressions and computed sex-specific measures of inequality and inequity. We explored the relationship between income and health services use separately by sex at end-of-life because women and men, on average, tend to use different levels of health care throughout the life course and die at different ages and of different causes. Thus, we aimed to investigate whether income-related inequities in health services spending also varied by sex.

We used linked administrative databases to study the income-related inequities in spending on four main facets of care: hospital care, general practitioner services, specialist services, and prescription drugs. In our analysis, we controlled for age, cause of death, sex, area of residence, and clinical complexity using standard health equity measures that facilitated comparison to previous equity studies from Canada, the U.S. and Europe.

## Methods

### Data

With permissions from relevant data stewards at the B.C. Ministry of Health Services and the College of Pharmacists of B.C., we obtained de-identified linked health data from Population Data B.C. and B.C. PharmaNet [19]. These databases cover all B.C. residents except those whose health care (and therefore health data) is under federal jurisdiction: status Indians, military personnel, and the Royal Canadian Mounted Police (under 5% of the total population). For all other individuals, the datasets contain demographic information, records of all non-emergency hospital admissions, records of all fee-for-service medical visits (general and specialist care), and all prescriptions filled outside of acute care hospitals (regardless of payer). All residents of B.C. receive universal, first-dollar public insurance for hospital care and for general and specialist medical services. Prescription drugs for B.C. residents are covered through a universal public drug plan with income-based deductibles, private employer or individually-sponsored insurance, and out-of-pocket payments (private payments represent approximately 55% of total drug spending in B.C.) [20]. Ethics approval for this study was obtained from the Behavioural Research Ethics Board at the University of British Columbia.

### Cohort

To define our cohort of decedents, we retrospectively identified deaths in the calendar years 2004, 2005, and 2006 using vital statistics data. We excluded deaths for those who were under 65 to further minimize the unobserved health differences within our decedent cohort. For the same reason, we excluded those who died of external causes, including accidents and suicides. We also excluded from our analysis decedents who did not live in B.C. for at least 275 days in the year before death to minimize misclassification bias relating to unobserved use and unmeasured health status. Finally, to reduce the extent to which use of medical services is undercounted, we excluded residents of local health areas wherein greater than 30% of all medical services are provided non-fee-for-service, and therefore fall outside of our dataset (representing ~0.5% of all deaths). Our final cohort included 58,820 decedents.

Dates of death in our datasets contained only year and month for privacy reasons. We therefore defined the 'year' prior to death to include the month of death plus the 12 preceding months.

### Variables

Our analysis focused on the amount of health care resources used, which we measured in terms of total cost of acute care services. For the year prior to each subject's death, we calculated the cost of non-emergency hospital care, fee-for-service care from general practitioners, fee-for-service care from specialist physicians, and prescriptions filled outside of acute care hospitals. Costs for prescription drugs were included regardless of coverage and included the pharmacist fees associated with each prescription filled. Costs for pharmaceuticals and fee-for-service medical care were based on actual fees and prices paid. However, because hospitals are block funded in Canada, the cost of hospital care was computed using standard costing methods for cases of differing resource intensity [8,21,22]. We ran analyses for five dependent variables: total acute costs (the sum of hospital, medical and prescription drug expenditures), and separately hospital, fee-for-service general practitioner, fee-for-service specialist care, and prescription drugs. All costs were inflation-adjusted to 2006 Canadian dollars.

Our independent variable of primary interest was household income percentile. For approximately 95% of our cohort, our income percentiles were based on household-specific income data for 2003 that was validated using Canada Revenue Agency data by the B.C. Ministry of Health Services. For the remaining sample, our income percentiles were based on average incomes reported in year-2003 tax returns by households across Census Dissemination Areas (comprising 400-700 persons) [23]. For regression analyses, we created dummy variables for each income quintile in order to allow for non-linearities in relationships between income and dependent variables. Incomes were re-grouped into sex-specific percentiles and quintiles for sex disaggregated analyses.

Based on diagnosis codes in medical and hospital records for the last year of life, we used Aggregated Diagnostic Groups (ADGs) of the Johns Hopkins ACG^® ^Case-Mix system to measure each decedent's general clinical complexity [24]. Each hospital record contains up to 25 diagnosis codes and each record of a fee-for-service medical visit contains one diagnosis code. The ACG system maps these diagnoses into 32 ADGs of varying clinical severity. Groups with the highest expected resource use are called major ADGs. We counted separately the number of major ADGs and the number of non-major ADGs for each individual.

We controlled for age at death using dummy variables representing 5-year age bands (e.g. 65 to 69), with a combined group for those 100 and older, as we have previously identified non-linear relationships between age and use of health care [25,26]. We accounted for whether an individual resided in a long term care facility or received palliative care based on program indicators from the drug claims database. We also controlled for the effects of urban/rural differences in resource availability and care use; to be considered urban, the majority of residents of the local area had to have resided in the dominant city (comprised of at least 25,000 residents). Finally, because cancer deaths are likely to result in distinct patterns of end-of-life care [27], and cancer and cardiovascular-related deaths account for the large majority of deaths and vary (seemingly symmetrically) with income within our sample, we controlled for cancer cause of death using vital statistics records. Because pooled decedent data (2004-2006) were used in order to increase sample size, we also controlled for year of death in all analyses.

### Statistical Analysis

In our regression analyses, we used generalized linear methods (GLM) using the log link and the gamma distribution, selected using standard methods for health cost data [28,29]. GLM allowed us to relax the strict normal error assumption of linear least squares regression (which right-skewed, zero clustered health expenditure data often violate) and avoid the complications of retransforming estimates to eliminate potential bias in non-linear or log-transformed least squares [30,31]. Because nearly our entire cohort had positive expenditures for the services examined, we used one-step GLM models.

We used concentration indexes (CIs) to measure the differences in spending on services by type, and horizontal inequity (HI) index measures to quantify inequities in spending unrelated to need [32,33]. In the context of the present study, a CI summarizes the extent to which the distribution of health care resources (costs) differs from uniformity across income groups (percentiles). A CI will be zero when resources are distributed equally across income groups, negative when resources are concentrated among those with low incomes, and positive when resources are concentrated among those with high incomes.

Some of the resource concentration captured by the CI may reflect income-related inequalities in health need. A measure of inequity (as opposed to inequality) must account for income-related differences in health status and thereby quantify how observed patterns of care deviate from the principle of 'equal treatment for equal need' [5]. To construct such a measure, the HI index [32], we first used regression analysis to predict the amount of health care we would have expected each individual to receive based on her or his age, sex, health status, and cause of death, holding other variables including income, nursing home residence, and region of residence at the sample mean. The predicted values from this analysis are estimates of the amount of health care resources that a decedent would have received if treated the same as the average person with the same need profile [33]. We used these values to compute need-predicted concentration indexes for each category of health spending.

We then computed the HI for expenditure for each service as the difference between the observed inequality in spending and the inequality in spending that would be predicted based on need. The HI thus measures how much of the observed inequality in the distribution of spending on health care is left unaccounted for by the unequal distribution of health-related need. The HI measure will be zero when health care spending is distributed across incomes in proportion to how health care need is distributed. A negative (positive) HI means that health care spending is more concentrated in lower (higher) income groups than health care need would predict. One can quantitatively interpret the HI as roughly the share of expenditure that would need to be transferred from the half of the distribution with greater than predicted spending to the other in order for spending to equitably reflect health need [34]. We reported both the CI that measures the income-related inequalities in actual health care expenditures and the HI. While we expected regression and equity analyses to generate similar findings, we discuss both: regression findings put our results in the context of previous end-of-life literature, while equity analyses allowed us to directly compare the degree of inequity measured, using HI estimates, to previous equity results.

## Results

Table 1 describes our study population by income quintile. Those with the lowest incomes were older, female, non-urban dwelling, more likely to live in a residential care facility, less likely to use the palliative care program, and had fewer diagnosed conditions than those with the highest incomes. Some of these differences by income (e.g., age and palliative care) are likely to be related to differences in cause of death: low-income decedents were more likely to die of cardiovascular and respiratory conditions but less likely to die of cancer than high-income decedents. Table 1 also lists rates of use, various use intensity measures, and mean per capita expenditure by service type. Use of hospitals, specialist services and prescription drugs were higher in higher income groups than for those of lower income, while nearly all decedents visited a general practitioner, regardless of income. Expenditures for each type of service examined were higher in the highest income group compared to the lowest.

**Table 1:** Description of study population, by income quintile

	**Income quintile**					
	**1 (lowest)**	**2**	**3**	**4**	**5 (highest)**	**Total**

N	11,764	11,764	11,764	11,764	11,764	58,820

Died 2004, %	34 (0.44)	34 (0.44)	34 (0.44)	33 (0.43)	31 (0.43)	33 (0.19)
Died 2005, %	33 (0.43)	34 (0.44)	33 (0.43)	32 (0.43)	33 (0.43)	33 (0.19)
Died 2006, %	33 (0.43)	33 (0.43)	34 (0.44)	34 (0.44)	35 (0.44)	34 (0.19)
						
Female, %	71 (0.42)	63 (0.45)	48 (0.46)	43 (0.46)	38 (0.45)	52 (0.21)
Age at death, mean	83.5 (0.09)	84.1 (0.07)	82.1 (0.07)	81.0 (0.07)	80.4 (0.07)	82.2 (0.03)
Urban resident, %	81 (0.24)	79 (0.25)	78 (0.26)	78 (0.25)	84 (0.20)	80 (0.11)
Major ADGs, mean count	2.6 (0.01)	2.7 (0.01)	2.8 (0.01)	2.8 (0.01)	2.9 (0.01)	2.8 (0.01)
Minor ADGs, mean count	5.8 (0.03)	6.1 (0.03)	6.2 (0.02)	6.2 (0.03)	6.3 (0.02)	6.1 (0.01)
Cancer cause of death, %	20 (0.37)	22 (0.38)	26 (0.41)	29 (0.42)	32 (0.43)	26 (0.18)
Cardiovascular cause of death, %	28 (0.41)	27 (0.41)	25 (0.40)	24 (0.39)	23 (0.39)	25 (0.18)
Respiratory cause of death, %	14 (0.32)	13 (0.31)	13 (0.31)	11 (0.29)	11 (0.29)	12 (0.14)
Residential care, %	28 (0.41)	26 (0.40)	22 (0.38)	20 (0.37)	18 (0.35)	23 (0.17)
Palliative care Rx coverage, %	11 (0.29)	12 (0.30)	15 (0.33)	17 (0.35)	21 (0.37)	15 (0.15)
						
**USE**						
Hospital, %	68 (0.43)	72 (0.42)	73 (0.41)	73 (0.41)	75 (0.40)	72 (0.18)
General practitioner, %	98 (0.14)	99 (0.10)	99 (0.10)	99 (0.10)	99 (0.09)	99 (0.05)
Specialist physicians, %	92 (0.25)	94 (0.22)	94 (0.21)	95 (0.21)	96 (0.19)	94 (0.10)
Prescription drug, %	86 (0.32)	86 (0.32)	86 (0.32)	88 (0.30)	90 (0.28)	87 (0.14)
Nights in hospital, mean	19 (0.28)	21 (0.29)	21 (0.30)	20 (0.28)	20 (0.29)	20 (0.13)
General practitioner contacts, mean	24.2 (0.18)	26.4 (0.18)	26.7 (0.18)	26.9 (0.18)	26.8 (0.18)	26.2 (0.08)
Specialist contacts, mean	17.4 (0.24)	19.1 (0.24)	20.9 (0.25)	22.7 (0.26)	25.6 (0.27)	21.1 (0.11)
Count of drug types (ATCL3), mean	8.14 (0.05)	8.38 (0.05)	8.42 (0.05)	8.59 (0.05)	8.91 (0.05)	8.49 (0.02)
Total days of prescriptions, mean	1428 (11.5)	1460 (11.3)	1442 (11.3)	1428 (11.0)	1447 (10.7)	1441 (5.0)
Number of prescriptions, mean	60 (0.82)	61 (0.83)	54 (0.71)	50 (0.63)	47 (0.59)	54 (0.33)
						
**EXPENDITURES ($2006)**						
Total	19,063 (238)	20,183 (239)	21,213 (254)	20,870 (243)	22,201 (268)	20,705 (111)
Hospital	15,165 (220)	16,035 (222)	16,787 (236)	16,249 (224)	17,111 (247)	16,269 (103)
General practitioners	949 (7.7)	1,050 (7.9)	1,076 (7.9)	1,071 (7.8)	1,059 (7.6)	1,041 (3.5)
Specialist physicians	1,380 (18.6)	1,460 (18.1)	1,674 (19.9)	1,803 (21.2)	2,072 (23.1)	1,678 (9.1)
Prescription drugs	1,526 (17.1)	1,593 (17.0)	1,639 (17.4)	1,726 (19.4)	1,939 (22.7)	1,685 (8.4)

ADGs = Aggregated Diagnostic Groups

Standard errors in parentheses

Table 2 lists the adjusted associations between income quintile and health care spending in the last year of life for sex-stratified and pooled models (which include sex as a covariate). These models adjust for age, health status, cause of death, use of long term care facilities, rural residence, and year of death (see the Appendix Table in Additional file 1 for full regression results). For comparison with previous end-of-life literature, the first column of results describes total health care spending. For all costs combined in both the sex-pooled and sex-disaggregated models, there was no statistically significant difference in total health care spending in the last year of life between the lowest, second lowest and middle income quintiles after other factors were accounted for. For women and in the sex-pooled, model total measured spending on care in the last year of life for those in the highest two income quintiles was slightly below (by 4-6%) those in the lowest income quintile. For men, there was no statistically significant difference.

**Table 2:** Regression results: adjusted relationship between income quintile and spending on health services in the last year of life for a cross-section of British Columbians in their last year of life, 2004-2006

**Total Population (n = 58,880)**					
	**All services combined**	**Hospital care**	**General practitioners**	**Medical specialists**	**Prescription drugs**
Income 1 (reference)	---	---	---	---	---
Income 2	0.024	0.009	0.050***	0.040**	0.072***
Income 3	0.006	-0.020	0.061***	0.055***	0.073***
Income 4	-0.040**	-0.091***	0.050***	0.065***	0.106***
Income 5	-0.037*	-0.115***	0.020*	0.137***	0.208***
					

**Females (n = 30,087)**					
	**All services combined**	**Hospital care**	**General practitioners**	**Medical specialists**	**Prescription drugs**
Income 1 (reference)	---	---	---	---	---
Income 2	-0.014	-0.051	0.005	0.017	0.055**
Income 3	-0.004	-0.030	0.032**	0.033	0.067**
Income 4	-0.058**	-0.111**	0.014	0.041	0.030
Income 5	-0.059**	-0.132***	-0.009	0.076***	0.122***
					

**Males (n = 28,013)**					
	**All services combined**	**Hospital care**	**General practitioners**	**Medical specialists**	**Prescription drugs**
Income 1 (reference)	---	---	---	---	---
Income 2	0.038	0.022	0.068***	0.068***	0.118***
Income 3	0.014	-0.015	0.096***	0.083***	0.151***
Income 4	-0.030	-0.096**	0.063***	0.104***	0.222***
Income 5	-0.026	-0.116***	0.022	0.200***	0.316***
					

Model adjusted for age, health status, cancer death, year of death, urban dwelling, residential care, and sex (sex-pooled model only). See Additional file 1 for all regression coefficients.

Regression models are GLM with log link (dependent variables thus modeled as log of expenditure in last year of life).

* p < 0.05, ** p < 0.01, *** p < 0.001

After other factors were accounted for, income gradients for spending during the last year of life differed significantly by type of health care. In sex-pooled results the gradient for hospital spending was negative whereas gradients for spending on general practitioner services, specialist services, and prescription drugs were generally positive. Because hospital spending represents, on average, 80% of total measured health costs in the last year of life, hospital and total spending regressions are fairly similar. Spending on hospital care was approximately 10% lower for decedents in the highest two income quintiles compared to the lowest income quintile.

In the sex-pooled regression analysis and separately for men the gradient in spending on general practitioner services among the last year of life was positive, but decreasing. Decedents in the middle income quintiles had 5-6% higher spending than those in the lowest quintile while decedents in the highest quintile had 2% higher spending than those in the lowest quintile. The income gradient for spending on general practitioner services was not significant for women.

After other factors were accounted for, spending on specialist medical care and prescription drugs during the last year of life was more positively correlated with income than spending on general practitioners, particularly among men. Spending on specialist care during the last year of life by males in the highest quintile was 20% higher than such spending by males in the lowest income quintile. Similarly, spending on prescription drugs by high-income men during their last year of life was approximately 30% higher than prescription drug spending by men in the lowest income quintile. For women, spending on specialist medical care and on prescription drugs during the last year of life was significantly higher among those in the highest income quintile compared to those in the lowest income quintile; however, the gradient across income quintiles was not significant for specialist care and non-linear for prescription drugs.

Table 3 lists the concentration indexes and horizontal inequity measures for hospital services, general practitioners, specialists and prescription drugs. For the sex-pooled model, resource use was concentrated in higher income groups, for all services examined, before adjusting for need (CI). After adjusting for need, spending on hospital care and general practitioner services was disproportionately concentrated in those of lower income (HI -0.031 and -0.006, respectively). While less dramatic than the concentration index measures, spending on specialist services and prescription drugs remained concentrated in those with higher incomes (HI 0.034 and 0.033). Across types of care studied, the degree of measured horizontal inequity in health care spending was greater for men than women, particularly for specialist medical care and prescription drugs, which are disproportionately concentrated in higher income decedents.

**Table 3:** Inequality and inequity in spending on hospital, general practitioners, specialists, and pharmaceuticals at end-of-life

	**Total Population**	
	
	**Inequality (CI)**	**Inequity (HI)**
	
Hospital	**0.017**	**-0.031**
General practitioner	**0.017**	**-0.006**
Specialist physician	**0.078**	**0.024**
Prescription drugs	**0.044**	**0.033**
	
	Females	
	
	Inequality (CI)	Inequity (HI)
	
Hospital	0.006	**-0.028**
General Practitioner	**0.009**	-0.006
Specialist physician	**0.062**	**0.022**
Prescription drugs	**0.040**	**0.020**
	
	Males	
	
	Inequality (CI)	Inequity (HI)
	
Hospital	0.002	**-0.031**
General practitioner	**0.015**	**-0.008**
Specialist physician	**0.057**	**0.030**
Prescription drugs	**0.065**	**0.055**

Bold values statistically significant at p < 0.05.

CI = Concentration Index, HI = Horizontal Inequity

## Discussion

Using population-based sources of administrative health care data, we found evidence of significant inequities in the need-adjusted distribution of health services at end-of-life. When health need is accounted for, decedents of higher income have higher expenditures than those of lower income for prescription drugs and specialists; for hospital services, spending was concentrated in those of lower income more so than the income distribution of need would predict. Spending on general practitioners showed a very small concentration in those of low income, although regression results highlighted a non-linear relationship between need-adjusted spending and income. For all types of services examined, inequities appeared more pronounced among male decedents than females.

Results of our regression analyses showing a negative relationship between income and total acute healthcare spending at end-of-life are consistent with previous analyses of total Medicare expenditures for sample decedent populations [14,15]. Yet, the results for total acute spending contrast a recent analysis of a full population of Stockholm decedents which found spending at end-of-life on the same suite of services to increase with income [17]. That analysis did not include detailed information on individual co-morbidities; our findings of a negative income relationship for overall end-of-life spending after need adjustment are largely influenced by accounting for health status, and allowing for a non-linear relationship between age at death (which varies with income) and spending. Sensitivity analyses in which we omitted health status controls (major and non-major ADGs) found no significant relationship between income and total measured health care spending (data available upon request). By using improved health status measures combined with full population data and allowing for flexible relationships between health care spending and covariates, our analysis improves on these previous end-of-life studies.

Using an end-of-life cohort for our equity analysis, our measures of horizontal inequity for hospital and medical services care were smaller than but similar to measures from survey-based studies of full European and North American populations [3,5,6]. Our results concerning inequities in end-of-life spending on hospital, general practitioner and specialist care are of the same direction, although generally larger, than those found using administrative data for the full population (including non-decedents) of B.C. in 2002 [8]. Results for specialist services were similar to those found using survey data from 2003. Hospital care results in that study were not significant for B.C.; however they found pro-poor inequities in hospital care for Canada as a whole [7]. While small sample sizes might have limited province-specific results in that study (their B.C. sample was 12,367), point estimates of HI suggested pro-rich inequities in use of inpatient care as measured by total nights in hospital.

Previous research that compared horizontal inequity in universally covered services (including hospital, GP and specialist services) to partially covered services (including pharmaceuticals) in Denmark found statistically significant inequities only for partially covered services, with amount spent concentrated in those with higher incomes [35]. Point estimates of horizontal inequity for hospital and specialist services, while insignificant, were similar in size to ours; however, the HI for pharmaceuticals was three times as large in the Danish study. Such differences are likely related to differences in public drug plans: in B.C., public coverage is based on household income and the amount spent on pharmaceuticals, while in Denmark public reimbursement is related only to the absolute amount spent on pharmaceuticals.

While a higher concentration of spending on specialists and pharmaceuticals by higher income decedents is likely not surprising, given other socio-economic differences that may make accessing such services easier for those of higher income, including education, private support systems, employer-based insurance and relative affordability of any out-of-pocket payments, it is perhaps more surprising to find the reverse income gradient for hospital services. Some of this may be substitution of hospital care for other health services. As research from the US suggests, low income is associated with a higher number of ambulatory care-sensitive condition admissions at the hospital level for Veterans Affairs patients [36]. Further research focusing on hospital care could illuminate how use of varying types and different intensities of end-of-life care within hospitals contributes to income-related inequities in spending.

Our results should be interpreted within the context of several notable limitations. First, we lack information on continuing care, whether provided in skilled nursing facilities or at home, both of which may substitute for some forms of hospital care at the end-of-life. Including publicly funded care would have potentially steepened the income gradient found in analysis of combined health services, as residence in care homes appears higher for lower income decedents (see Table 1); however, previous research suggests an unclear relationship between income and continuing care use [37-39].

A second limitation of our analysis relates to our income measure. Ideally, we would have had income values for each decedent for the year preceding their death. However, given the stability of income over time and the short period of our analysis, misclassification from using 2003 incomes was likely minimal.

We analysed spending on each health service, which acts as a proxy of resource use intensity, but does not measure the quality of service. A quality adjustment to dollars spent might have produced a stronger income gradient in medical services. A study of home visits by family practitioners to end-of-life cancer patients in Nova Scotia found that while nearly all patients had contact with a family practitioner in their last six months of life, those living in lower income areas were less likely to receive home visits compared to high and middle income area residents [40]. Quality adjustments may also have mitigated income findings if those receiving more care in terms of dollars spent were somehow getting care of lower quality or excessive levels of care. In other words, because we lack a measure of "optimal" or "appropriate" care, our findings of a pro-poor distribution in hospital spending at end-of-life may reflect some overuse of such care by persons with low incomes. Similarly, the pro-rich findings with respect to specialist medical care and prescription drugs might represent over-provision of such care to wealthier patients. However, a recent study of Canadian populations suggests that including a measure of unmet need in equity analyses, allowing for heterogeneity in the level of optimal care, did not affect equity findings [41]. The robustness of inequity findings suggests that identifying potential systemic reasons for the observed patterns of inequity is important in designing policies to address these patterns.

## Conclusions

This paper is the first to our knowledge to comparatively measure the level of inequity in health care spending for decedents across various types of care using standard health equity measures. It is also the first to comparatively examine inequities in spending by type separately for men and women decedents. We found higher spending on specialist services and pharmaceuticals at end-of-life for those with higher incomes, which mirrors pro-rich patterns of spending and use in non-decedent populations. This suggests continuity of income inequities in health care use over the life course and the potential lock-in of care inequities over time. The concentration of hospital spending among those of lower incomes may be reflective of trade-offs in care between specialist services, general practitioners, and hospitals, or substitution of hospital care for home-based health and social care [42], that may signal inefficiencies. While our measures of inequity, put in international context, are relatively small, findings of inequities in a population for which unobserved health differences are minimized confirm the presence of systemic inequities in access to care.

## Competing interests

The authors declare that they have no competing interests.

## Authors' contributions

All authors contributed to project conception and study design. CMC was responsible for data analysis, interpretation of results, and was the principal writer of the manuscript. GEH contributed to interpretation of results and manuscript preparation and revision. SGM was responsible for the acquisition of the data and contributed to interpretation of results and manuscript preparation and revision. All authors approved the final version of the manuscript.

## Supplementary Material

Additional file 1 **Table S1**. Full regression results - Adjusted relationship between spending on health services in the last year of life and determinants for a cross-section of British Columbians in their last year of life, 2004-2006. The Appendix Table provides expanded results for regressions from Table 2 (including non-income coefficient estimates). Click here for file
