# Supplementary material for: Income inequities in end-of-life health care spending in British Columbia, Canada: A cross-sectional analysis, 2004-2006
Source: Int J Equity Health. 2011 Mar 16;10:12. doi: 10.1186/1475-9276-10-12 (PMC3070644; doi:10.1186/1475-9276-10-12)
Supplement: Additional file 1 — Table S1. Full regression results - Adjusted relationship between spending on health services in the last year of life and determinants for a cross-section of British Columbians in their last year of life, 2004-2006. The Appendix Table provides expanded results for regressions from Table 2 (including non-income coefficient estimates). [file 1475-9276-10-12-S1.DOCX]

#### Table S1: Full regression results: Adjusted relationship between spending on health services in the last year of life and determinants for a cross-section of British Columbians in their last year of life, 2004-2006

|  | Total Population (n=58,880) | | | | |  | Females (n=30,087) | | | | |  | Males (n=28,013) | | | | |
| --- | --- | --- | --- | --- | --- | --- | --- | --- | --- | --- | --- | --- | --- | --- | --- | --- | --- |
|  | All | Hospital | GPs | Spec. | Drugs |  | All | Hospital | GPs | Spec. | Drugs |  | All | Hospital | GPs | Spec. | Drugs |
| Income 1 (ref.) | --- | --- | --- | --- | --- |  | --- | --- | --- | --- | --- |  | --- | --- | --- | --- | --- |
| Income 2 | 0.024 | 0.009 | 0.050*** | 0.040** | 0.072*** |  | -0.014 | -0.051 | 0.005 | 0.017 | 0.055** |  | 0.038 | 0.022 | 0.068*** | 0.069*** | 0.118*** |
| Income 3 | 0.006 | -0.020 | 0.061*** | 0.055*** | 0.074*** |  | -0.004 | -0.030 | 0.032** | 0.033 | 0.067** |  | 0.014 | -0.015 | 0.096*** | 0.083*** | 0.151*** |
| Income 4 | -0.040** | -0.091*** | 0.050*** | 0.065*** | 0.106*** |  | -0.058** | -0.111** | 0.014 | 0.041 | 0.030 |  | -0.030 | -0.096** | 0.063*** | 0.104*** | 0.222*** |
| Income 5 | -0.037* | -0.114*** | 0.020*** | 0.137*** | 0.208*** |  | -0.059** | -0.132*** | -0.009 | 0.076*** | 0.122*** |  | -0.026 | -0.116*** | 0.022 | 0.200*** | 0.316*** |
|  |  |  |  |  |  |  |  |  |  |  |  |  |  |  |  |  |  |
| Age 65-69 | 0.006 | -0.028 | 0.065*** | 0.032 | 0.111*** |  | 0.006 | -0.044 | -0.049* | 0.034 | 0.126*** |  | 0.003 | -0.023 | -0.077*** | 0.027 | 0.104*** |
| Age 70-74 (ref.) | --- | --- | --- | --- | --- |  | --- | --- | --- | --- | --- |  | --- | --- | --- | --- | --- |
| Age 75-79 | -0.049** | -0.037 | 0.004 | -0.100*** | -0.092*** |  | -0.095** | -0.093 | -0.026 | -0.157*** | -0.110*** |  | -0.018 | -0.001 | 0.022 | -0.059** | -0.077** |
| Age 80-84 | -0.128*** | -0.067 | 0.022* | -0.282*** | -0.320*** |  | -0.132*** | -0.068 | -0.004 | -0.326*** | -0.262*** |  | -0.136*** | -0.079* | 0.037** | -0.251*** | -0.372*** |
| Age 85-89 | -0.175*** | -0.102*** | 0.027* | -0.475*** | -0.432*** |  | -0.214*** | -0.160*** | -0.015 | -0.521*** | -0.361*** |  | -0.140*** | -0.045 | 0.063*** | -0.442*** | -0.509*** |
| Age 90-94 | -0.268*** | -0.174*** | 0.016 | -0.684*** | -0.605*** |  | -0.303*** | -0.224*** | -0.021 | -0.743*** | -0.557*** |  | -0.232*** | -0.118** | 0.051** | -0.629*** | -0.650*** |
| Age 95-99 | -0.364*** | -0.265*** | -0.004 | -0.847*** | -0.771*** |  | -0.425*** | -0.340*** | -0.047* | -0.931*** | -0.767*** |  | -0.261*** | -0.136* | 0.047 | -0.721*** | -0.714*** |
| Age 100+ | -0.610*** | -0.542*** | -0.117*** | -1.102*** | -1.132*** |  | -0.676*** | -0.602*** | -0.158*** | -1.234*** | -1.106*** |  | -0.410*** | -0.310* | -0.048 | -0.789*** | -1.074*** |
| Female | -0.007 | -0.033* | 0.022*** | -0.076*** | 0.188*** |  |  |  |  |  |  |  |  |  |  |  |  |
| Major ADGs | 0.387*** | 0.513*** | 0.156*** | 0.341*** | 0.060*** |  | 0.394*** | 0.546*** | 0.164*** | 0.351*** | 0.070*** |  | 0.381*** | 0.484*** | 0.146*** | 0.332*** | 0.050*** |
| Minor ADGs | 0.183*** | 0.228*** | 0.161*** | 0.137*** | 0.083*** |  | 0.187*** | 0.239*** | 0.160*** | 0.140*** | 0.088*** |  | 0.177*** | 0.213*** | 0.161*** | 0.133*** | 0.075*** |
| Cancer death | 0.076*** | 0.100*** | 0.212*** | 0.187*** | 0.062*** |  | 0.095*** | 0.130*** | 0.222*** | 0.236*** | 0.048*** |  | 0.057*** | 0.075*** | 0.203*** | 0.140*** | 0.078*** |
| Long Term Care | -0.150*** | -0.351*** | -0.061*** | -0.177*** | 0.401*** |  | -0.178*** | -0.423*** | -0.078*** | -0.101*** | 0.371*** |  | -0.094*** | -0.227*** | -0.026* | -0.293*** | 0.451*** |
| Rural | 0.006 | 0.039 | 0.166*** | -0.024*** | 0.035 |  | 0.012 | 0.066 | 0.157*** | -0.264*** | 0.031 |  | 0.002 | 0.018 | 0.175*** | -0.225*** | 0.042 |
| Died 2005 | -0.009 | -0.017 | -0.047*** | -0.004 | 0.057*** |  | 0.006 | -0.002 | -0.049*** | 0.017 | 0.080*** |  | -0.023 | -0.031 | -0.045*** | -0.026 | 0.031 |
| Died 2006 | -0.054*** | -0.094*** | -0.077*** | 0.004 | 0.095*** |  | -0.049** | -0.010*** | -0.084*** | 0.024 | 0.120*** |  | -0.059*** | -0.087*** | -0.069*** | -0.016 | 0.065*** |
| Constant | 7.572*** | 6.625*** | 5.313*** | 5.618*** | 6.636*** |  | 7.568*** | 6.512*** | 5.378*** | 5.514*** | 6.745*** |  | 7.596*** | 6.748*** | 5.304*** | 5.658*** | 6.687*** |
|  |  |  |  |  |  |  |  |  |  |  |  |  |  |  |  |  |  |

Reference groups: Lowest Income quintile, Age 70-74, Male (for total results), No Major or minor ADGs, Non-Cancer death, no residential care, died in 2004, non-rural resident.

Regression models are GLM with log link (dependent variables thus modeled as log of expenditure in last year of life).

* p<0.05, ** p<0.01, *** p<0.001
